# Active and Passive Electro-Optical Sensors for Health Assessment in Food Crops

**DOI:** 10.3390/s21010171

**Published:** 2020-12-29

**Authors:** Thomas Fahey, Hai Pham, Alessandro Gardi, Roberto Sabatini, Dario Stefanelli, Ian Goodwin, David William Lamb

**Affiliations:** 1 School of Engineering, RMIT University, Melbourne, VIC 3000, Australia; s3163948@student.rmit.edu.au (T.F.); s3612607@student.rmit.edu.au (H.P.); alessandro.gardi@rmit.edu.au (A.G.); 2 Food Agility CRC Ltd., 81 Broadway, Melbourne, NSW 2007, Australia; dario.stefanelli@dpird.wa.gov.au (D.S.); ian.goodwin@agriculture.vic.gov.au (I.G.); dave.lamb@foodagility.com (D.W.L.); 3 Manjimup Centre, Department of Primary Industries and Regional Development, Western Australia, Private Bag 7, Manjimup, WA 6258, Australia; 4 Agriculture Victoria, Tatura, VIC 3616, Australia

**Keywords:** agriculture, sensor, electro-optics, remote sensing, fluorescence, multispectral, hyperspectral, laser, food crop, LIDAR, spectroscopy, disease detection, heath assessment, artificial intelligence, machine learning, precision agriculture

## Abstract

In agriculture, early detection of plant stresses is advantageous in preventing crop yield losses. Remote sensors are increasingly being utilized for crop health monitoring, offering non-destructive, spatialized detection and the quantification of plant diseases at various levels of measurement. Advances in sensor technologies have promoted the development of novel techniques for precision agriculture. As in situ techniques are surpassed by multispectral imaging, refinement of hyperspectral imaging and the promising emergence of light detection and ranging (LIDAR), remote sensing will define the future of biotic and abiotic plant stress detection, crop yield estimation and product quality. The added value of LIDAR-based systems stems from their greater flexibility in capturing data, high rate of data delivery and suitability for a high level of automation while overcoming the shortcomings of passive systems limited by atmospheric conditions, changes in light, viewing angle and canopy structure. In particular, a multi-sensor systems approach and associated data fusion techniques (i.e., blending LIDAR with existing electro-optical sensors) offer increased accuracy in plant disease detection by focusing on traditional optimal estimation and the adoption of artificial intelligence techniques for spatially and temporally distributed big data. When applied across different platforms (handheld, ground-based, airborne, ground/aerial robotic vehicles or satellites), these electro-optical sensors offer new avenues to predict and react to plant stress and disease. This review examines the key sensor characteristics, platform integration options and data analysis techniques recently proposed in the field of precision agriculture and highlights the key challenges and benefits of each concept towards informing future research in this very important and rapidly growing field.

## 1. Introduction

**Figure 1:**
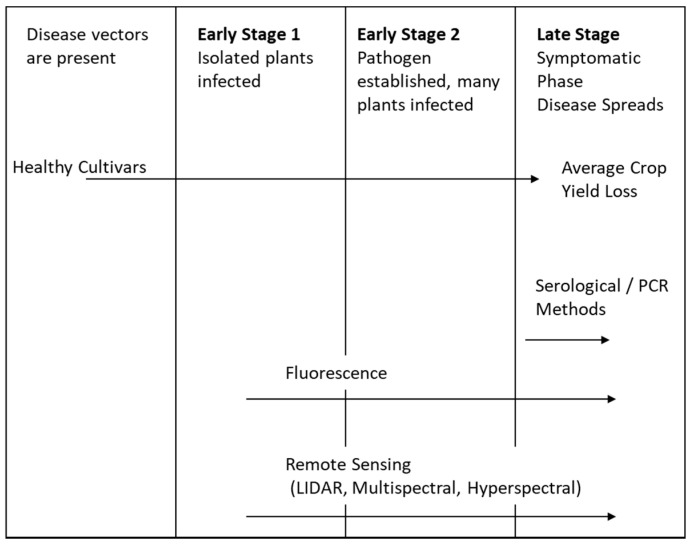
Disease spread and stages of sensor intervention. Adapted from [1].

Crop yield losses because of biotic and abiotic stresses affect roughly 10–30% of global food production [1,2,3]. With an increasing world population and a variety of factors reducing land and water resources, the answer to food insecurity is to increase crop yields [2,4]. Timely disease identification and quantification inform management decision-making to take preventative actions to reduce yield loss. Measurements on plants also allows for crops’ sensitivity and resistance to disease to be evaluated in relation to plant breeding, including the categorization of plant stresses. The use of pesticides as a protective measure can be excessive and expensive. Apart from the cost to production, the use of pesticides comes with an increased risk of toxic residue on crops and to the surrounding environment and ecosystems, as well as health implications for agriculture workers [4]. To increase production cost efficiency and yield, management need the information to be able to identify and quantify disease and pest epidemics accurately and at the earliest possible stage. This allows for contained, localised treatment, reducing the use of pesticides and potential yield losses. There are a variety of sensors and methodologies that aim to provide biotic (disease) and abiotic (nutrition) plant stress detection to farmers. These include in situ sampling techniques featuring fluorescence spectroscopy, visible and infrared spectroscopy and fluorescence imaging, as well as remote sensing (RS) techniques, such as multispectral imaging (MSI), hyperspectral imaging (HSI), thermography and light detection and ranging (LIDAR). The full range of early disease detection techniques is represented in Figure 1, categorised according to the stages of possible detection. The role of proximal and remote sensors in precision agriculture is discussed in relation to plant photosynthesis, phenotyping and soil quality. In addition, the key characteristics that define sensor performance, such as wavelength selection, classification indices, data analysis methods and platform integration, are explored.

## 2. Background

Traditionally, plant disease detection has been conducted through manual field surveys. These visual disease assessment practices have been criticized due to a large subjectivity in the diagnosis and reliance on clearly visible symptoms often only present in late-stage disease spread [1]. In situ sampling methods have demonstrated high levels of accuracy at the leaf level, with a range of handheld devices commercially available. However, in situ methods can be invasive, slow, costly and not a good spatial indicator of disease spread. The use of RS technologies such as MSI, HSI and LIDAR, implemented on a variety of platforms, has emerged to offer more objectivity in plant pathology analysis. RS techniques have been shown to be an effective noninvasive tool in determining the spatial distribution of diseases at the canopy level, particularly across large areas at low cost [5]. The non-destructive, noninvasive measurements obtained by RS can be done repeatedly and processed quickly, providing more objectivity in disease identification and quantification than a visual assessment. Drones are widely employed in precision agriculture, crop management and farming. A large variety of sensors (including various visual, multispectral and hyperspectral cameras, as well as LIDAR) are increasingly used on drones to carry out extensive surveys of crop conditions. The advantages of drones in agriculture are enhanced yields, time and cost savings, investment returns, flexibility, Geographic Information Survey (GIS) mapping integration and imaging crop conditions. Novel RS techniques like LIDAR are emerging to be the next generational leap forward in precision agriculture, with platforms exploiting LIDAR 3D scanning/shape profiling as well as more sophisticate techniques.

RS is generally defined as the measurement of objects at remote distances to acquire useful information indirectly by radiance or irradiance of electromagnetic energy from the surface of the Earth [6]. RS techniques are opposite to proximal sensing, which measures spectral features obtained by handheld or contact devices. Information on the scanned object, e.g., plant health, is retrieved by processing and analysing the acquired raw data and some additional environmental conditions. RS can detect plant conditions and analyse the spatial degree and patterns of plant characteristics using a noncontact method. Electro-optical sensors are typically differentiated between active and passive depending on whether energy is emitted from the device or not: In active sensors, signals are emitted, and their reflection/backscattering is measured, whereas in passive sensors, ambient irradiance such as solar radiation is exploited to detect the phenomenon of interest. Active RS instruments include Radar and LIDAR, while passive sensors include various detectors and imaging cameras which measure the reflected solar radiation in the wavelength regions of the visible spectrum (VIS: 400–700 nm), near-infrared (NIR: 700–1100 nm), shortwave infrared (SWIR: 1100–2500 nm) and thermal infrared (TIR: 3–15 µm) in the electromagnetic spectrum.

Most of the literature in the early detection of plant disease had focused on MSI and HSI techniques due to the maturity and implementations of these technologies. MSI and HSI have been applied to many kinds of crops to detect diseases. These two methods concentrate on the differences in the spectral signatures of infected and healthy plant leaves. For some crop diseases, early detection is important for farmers to yield the products economically and efficiently. As there are typically no visible symptoms at early stages of most diseases and fungi located on abaxial leaves, most visible sensors cannot detect early stages of diseases because the signatures are not sufficiently differentiated.

### 2.1. Plant Biology in Relation to Sensors

Plant health monitoring for precision agriculture encompasses the classification and quantification of the disease. Classification can be divided into two levels of increasing complexity: Detection and identification [1]. Detection is the distinction of healthy from unhealthy, and identification is the diagnosis of a specific disease or symptom. Quantification defines the spread and severity of the disease. The classification of plant diseases and stress is based on abiotic and biotic factors, shown in Table 1, and how they relate to plant physiological processes [7].

Sensors’ sensitivity to these various factors determine the effectiveness and accuracy of disease classifications to the extent of pre-symptomatic detection, as demonstrated in the literature [8]. Physiological changes in plants subject to biotic factors often result in similar symptoms [9,10]. Abiotic factors can also cause similar physiological symptoms and interact with the biotic factors, highlighting the difficulty in diagnosis and advocating the use of a multi-sensor approach, unlike the single sensor studies commonly found in the literature [11]. Plant health monitoring sensors mostly rely on detecting deviations in the leaf or canopy reflectance in the visible (VIS, 400 nm to 700 nm), near-infrared (NIR, 700 nm to 1100 nm) and shortwave infrared (SWIR, 1100 nm to 2500 nm) spectra. The spectral properties of leaves in these bands are the result of how the plant absorbs, reflects, emits, transmits and fluoresces under sunlight [11,12,13,14,15].

### 2.2. Wavelength Bands

**Figure 2:**
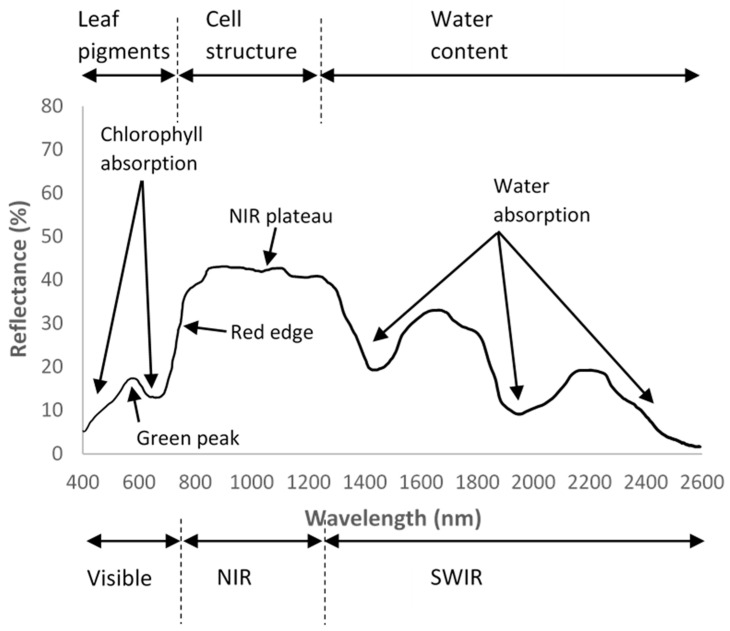
Generalized spectral reflectance profiles of vegetation. Adapted from [16,17].

The use of pigmentation as a plant health indicator is extensively used by in situ sampling methods, as well as some RS applications, as they directly relate to the photosynthetic and physiological properties. Electromagnetic radiation wavelengths are absorbed by pigments, water and biochemicals, resulting in a reduction of reflectance in corresponding spectral regions (Figure 2).

From the deviations in the visible and reflected infrared spectrum, plant health can be correlated and related back to abiotic and biotic stresses. In the visible infrared (VIS) between 400–700 nm, the reflectance is primarily correlated to the photosynthetic pigments like chlorophyll, (a and b), xanthophylls, anthocyanins and carotenoids [18]. Broadly, chlorophyll content is main source of interest in the VIS. Identification of the ‘green peak’ between roughly 500–700 nm and ‘chlorophyll well’ in the red band are used to detect plant stresses as shown in Figure 2. Between the VIS and near-infrared (NIR), the reflectance per wavelength spikes, called the red edge, which is a feature common to all green leaves and a useful plant health monitoring marker. In the NIR, the reflectance is related to the leaf tissue structure. In the shortwave infrared (SWIR), the spectrum reflectance is dominated by water absorption [19,20].

### 2.3. Plant Breeding

The assessment of plant’s resistance to stress is very desirable from a breeding perspective. Traditional methods of plant breeding are complex, extremely time-consuming and labour-intensive [21]. The selection of resistant cultivars is dependent on identifying the relevant plant traits through genotyping and phenotyping methods, although this method is also costly and slow, hence referred to as the phenotyping bottleneck [22]. The use of optical imaging methods as a new phenotyping method is suggested as an innovative technique to overcome the phenotyping bottleneck and improve breeding [22,23]. The phenotyping of soybeans has been successfully demonstrated using fluorescence spectroscopy, demonstrating the benefit and accuracy of optical, non-destructive methods [24]. The use of thermography is promoted as a useful method to improve phenotyping for drought adaption in maize [25]. Emerging techniques of extracting phenotypic parameters through LIDAR shape profiling have been developed using the leaf area density and height as key measurements [26]. Used in parallel with a thermal sensor, further insight into the plant health can be gained by established spectral reflectivity indicating desirable phenotypic parameters. Similarly, LIDAR shape profiling is used to assess to the canopy and above-ground biomass as part of a high-throughput phenotyping platform, delivering a non-destructive method and high correlation between the above-ground biomass and the LIDAR predicted volume and canopy height and providing vertical analysis of the biochemical properties, as explored with HSI LIDAR fusion by the authors of [27,28]. The analysis of LIDAR’s application to phenotyping is less mature: While acknowledging the variety and depths of information supplied by LIDAR in a short time frame at a low cost, particularly in relation to plant structure, the authors of [29] warned of inherent flaws in the methodology, the susceptibility of the technique to laser obscuration, inefficiencies in retrieving physiological traits and incomplete plant representation. These factors suggest that, in phenotyping, a LIDAR sensor will need to be paired with a complimentary hyperspectral sensor to overcome the limitations.

### 2.4. Soil Monitoring

Beyond health monitoring, RS techniques in fusion with GPS and GIS are used to monitor soil-related stressors and assist with land management. Crop yield can be effectively related to the soil conductivity from which the electrical conductivity can be projected from bands in VIS at 498 nm, 501 nm, 600 nm, 603 nm, 636 nm, 639 nm, 642 nm, 666 nm and 669 nm and in the NIR at 738 nm, 741 nm, 744 nm and 747 nm [16,30,31]. The information in these narrow bands is suited for HSI sensor detection gathered before seeding and can be correlated to soil properties such as moisture content, fertility, salinity, productivity estimates and other soil chemical properties. Measurements conducted during the growth season allow the detection of biotic and biotic stresses that impact crop yield, as discussed. Leaf area index (LAI) is one of the main variables used as an indicator of crop growth and yield as it correlates to the canopy reflectance. HSI has been validated by recent studies as preferable in LAI measurement to MSI as narrow band indices provide a better evaluation of LAI than in the broad range [17,32].

Increasingly, studies are investigating RS applications for soil analysis utilizing MSI and HSI. Notably, using a UAV platform and HSI, a study demonstrated a method for field-scale soil salinity monitoring, a major indicator of soil degradation and arable land loss, and outperformed the analysis conducted using satellite based MSI [33]. UAV-based HSI to determine soil moisture content as a key indicator of nutrient potential, on the other hand, highlights the emerging focus on artificial intelligence (AI) algorithms such as the random forest method and extreme learning as a means of soil moisture content estimation on a regional scale [34].

The practices of reducing soil erosion, enhancing water management and improving crop yields are crucial concerns in conservation agriculture. Three crop management practices—lowest possible soil disturbance, permanent soil cover and crop rotations—are proposed by conservation agriculture based on the Food and Agriculture Organization (FAO) definition [35]. The fertility of cultivated soils is related to the organic matter (OM) contents. Soil organic carbon (OC) content is forecasted by reflectance measurements, which are carried out in the lab with dried soil samples or in the field utilising spectroradiometers or satellite imaging. Remote sensors attached to drones can capture spatial images at specific altitudes to forecast soil OC on large-scale farmlands with bare soil before sowing season. For example, a drone integrated with infrared and visible light sensors captured aerial images to discover soil degradations by studying the appearance of gullies in the soil [36]. This was carried out by comparing the updated images with a set of historical images and satellite photographs. A fine spatial resolution image was used to quantify the area of soil erosion through a slow and cost-effective process. Emerging techniques are used to collect topographic data by drones to measure the quantity of soil volumes reduced via erosion. Terrestrial laser scanning (TLS) and images from drones and ground-based sensors are fused to obtain topography exploiting Structure from Motion (SfM) processing. Digital surface models obtained with each of these methods are subtracted from an interpolated pre-erosion surface model to evaluate the volumetric soil loss.

### 2.5. Classification Indices

The relationship between the collected data and classification of plant disease is usually characterized by a vegetation index (VI), as outlined in Table 2, which highlights a specific change in the spectral reflectance to distinguish between healthy leaves and those subject to abiotic and biotic stresses. Similarly, the use of hyperspectral vegetation indices specific to HSI was proposed [37].

### 2.6. Bidirectional Reflectance Distribution Function Measurement

The plant health assessment from spectroscopic sensors focuses on correlating the reflectance to photosynthesis to assess health. Therefore, the plant health assessment heavily relies on being able to take accurate measurements from the leaves. Understandably, in RS, the reflectance of the leaf can be influenced by the angle of leaf relative to the sensor, the ambient lighting spectrum and the intrinsic surface characteristics of the leaf itself. Inherently, the viewing angle and angle of illumination affect the brightness of the canopy, and subsequently, the crop identification, LAI and disease detection. The use of the canopy’s bidirectional reflectance distribution function (BRDF) can help to analyse the measurements made at non-optimal angles considering the surface characteristics of leaf [38]. The BRDF is a measurement of the ratio of radiance from a small beam of radiation compared to the incident flux density [38].

**Figure 3:**
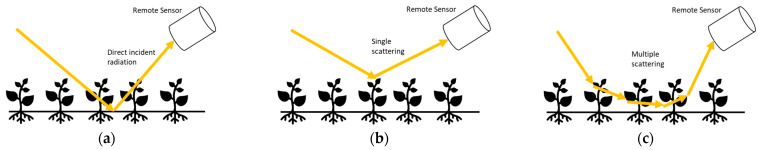
In-field scattering: (**a**) Unscattered; (**b**) Single scattering; and (**c**) Multiple path scattering. Adapted from [40].

In practise, the BRDF model is comprised of an equation correlating the surface physical properties to the observed reflectance as the light is subjected to scattering due to the canopy structure as depicted in Figure 3 [39]. The radiance is a function of the viewing and illumination angles and the incident flux density. The relationship between the BRDF of a canopy (ρ(Ω, Ω′) is the radiance, I(Ω), observed from direction (Ω), to the incident irradiance, *F_t_* [38].

(1) $$\rho \left(\Omega ,\;\Omega \prime \right)=\frac{I\left(\Omega \right)}{F_{t}}$$

Variables exist in the canopy, including how the plants shade the leaves and how the soil affects the reflectance. In fact, the leaves and soil themselves have their own BRDF, transmittance distribution and scattering characteristics, which has led to a generalised model for determining the BRDF for different canopies, mostly through geometric assumptions and Beer’s law relationship considering the interception of light for photosynthesis [38]. BRDF models are desirable because they are a compact means of storing and analysing large volumes of material, angular and spectral sampling data. The BRDF models are then responsible for interpolating often incomplete data to inform a BRDF prediction and for linking the BRDF to physical attributes such as the LAI [40].

## 3. Proximal Sensors

**Figure 4:**
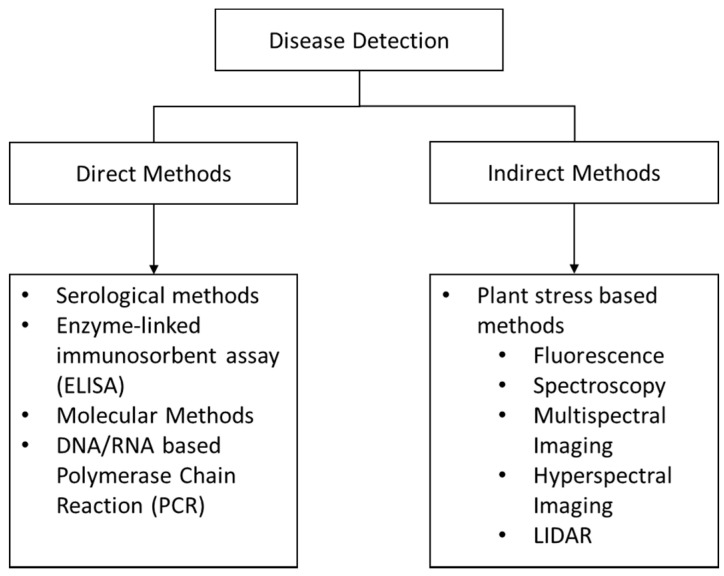
Disease detection methods. Reproduced with permission from Elsevier [41].

The most common way to assess plant health involves proximal sensors and destructive testing. Proximal sensors typically have high rates of classification accuracy but are time-consuming and do not encompass spatial information, and disease detection typically only occurs toward the late-stage, symptomatic spread of the disease. As Figure 4 shows, the sensors can be divided into direct and indirect methodologies to detect plant diseases.

### 3.1. Traditional Molecular Methods

A variety of molecular methods have been used for disease detection in plants. They are invasive, slow, proximal methods which are ill-suited to large field applications. A small overview is given below.

#### 3.1.1. Serological Assays

Serological assays are an early disease detection technique that are used to detect viruses. As the most prevalent assay, an enzyme-linked immunosorbent assay (ELISA) can detect bacteria at 100 Colony Forming Units (CFU) per mL^−1^ depending on the sample organism [1,42,43]. The advantage of serological assays is the range of diseases, bacteria and fungi that can be detected. The technique is disadvantaged by expense, destructive testing, low sensitivity, low detectability of diseases and storage of antibodies leading to contamination [44].

#### 3.1.2. Nucleic Acid-Based Methods

Nucleic acid-based methods are DNA- or RNA-based detection methods that generally rely on polymerase chain reaction (PCR) variant techniques [1]. An example of one of these methods is quantitative PCR (qPCR), a methodology that needs a sample to extract DNA, which then needs to be stored, amplified and replicated [1]. The technique fundamentally introduces errors as a result of the complexity of the methodology [45]. The unfeasible nature of large-scale testing can lead to false negatives due to the nonuniform spread of disease in plants, thereby creating a misinformed projection of plant disease spread. These techniques are advantageous in comparison to serological assays, with higher accuracy, faster detection times (within minutes, and lower cost [46]. Whereas molecular techniques are specific and efficient, they are disadvantaged by uneven disease distribution, poor sensitivity in certain materials and small sample sizes, which may not capture the true extent of the situation. PCR-based methods lack suitability for in-field operation and are also expensive and time-consuming [44,45]. These techniques are contrasted by the evolution of RS in precision agriculture applications as they inherently overcome the drawbacks of PCR-based and visual inspection methodologies.

### 3.2. Fluorescence Spectroscopy

Fluorescence spectroscopy is considered a cost-effective, fast and sensitive method [47]. The principles of fluorescence spectroscopy are based on the leaf properties reacting to light. When light is absorbed by the leaf, three processes occur in competition: Photochemistry (photosynthesis PSII), heat dissipation and re-emitted fluorescence [12]. Since these processes occur competitively, an increase in one of these processes causes a decrease in the other one or two. Using this principle, changes in the chlorophyll reflectance are monitored by handheld fluorometers, with deviations correlated to plant stress. Fluorescence spectroscopy uses an artificial light source to initiate the electron movement, which, in turn, generates luminescence [44]. The relative intensity of the reflected light is then indirectly related to plant diseases.

Fluorescence is particularly sensitive to external light, impacting in-field applications [48]. However, the introduction of Pulsed-Amplitude Modulation (PAM) artificial excitation light helps filter ambient light from the measured chlorophyll fluorescence. Fluorescence sensors are also critiqued as inefficient in detecting asymptomatic diseased leaves [45], are sensitive to leaf auto-fluorescence [49] and decrease control over the spread of a disease. Table 3 gives an overview of some of the research conducted using fluorescence spectroscopy in the detection of plant diseases.

## 4. Remote Sensing

**Figure 5:**
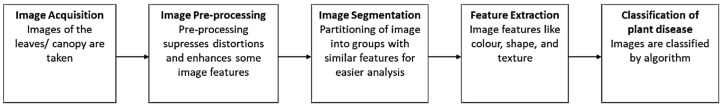
Methodology of spectral image analysis. Adapted from [44].

Emerging RS techniques rely on detecting anomalies in photosynthetic parameters. These alterations in pigment, chemical concentrations, nutrient, water uptake, cell structure and gas exchange can then be observed by the subsequent change in reflectance characteristics of the leaf or canopy [9]. The anomalous behaviour is then related back to a biotic or abiotic stress. It must be emphasised that the observed and quantified changes in spectral reflectance have an indirect relationship with the plant stressors. The data gleaned from the sensors are typically compared using one of the many vegetation indices and undergo significant data analysis to be classified between healthy and unhealthy, and between specific diseases. Each extra step invariably introduces uncertainty into the methodology. The results in the literature are highly nonuniform and difficult to compare quantitively as the research is specific to a crop and disease combination and subject to external factors. A unique spectral signature associated with each biotic or abiotic stress would enable a direct causal link between reflectance and for example water stress (abiotic). However, the absorption qualities of pigments and their overlap, for example, are affected by external elements, such as the leaf internal structure, water content and surface properties. Therefore, no single wavelength is unique to a single pigment concentration [9,57]. The lack of success in this approach has led researchers to use correlation analyses to define distinctive pathogen-specific spectral signatures, including a spectral index and ratio with discriminant analyses [9,58]. Conclusive optimal spectral signatures are clearly unsupported by the literature. However, the same studies still show good agreement with the sensitivity of certain spectral regions with high absorptions, corresponding to abiotic and biotic factors like pigmentation [57]. A generalised approach to plant health monitoring using sensors (in this case, hyperspectral) is shown in Figure 5.

Parametric analyses of spectral signatures of diseased plants are not common. Instead, researches have, for the most part, implemented nonparametric methods such as the Principal Component Analysis, Support Vector Machines, Cluster Analysis, Partial Least-Squares and Neural Networks [49]. A sample of these techniques is presented for each method: Fluorescence is presented in Table 3; with MSI, and HSI, presented below. Overall, a comparison of thermal, fluorescence and HSI advocates for a multi-sensor data fusion approach to plant health monitoring [59]. A particular study investigating head blight on wheat identified the major benefits and drawbacks of each system and further investigated the specific combinations of the sensors. Thermography-based sensors visualised the temperature differences between crops affected by biotic and abiotic stressors using IR in the 7.5–12 μm wavelength band. Chlorophyll fluorescence-based techniques in the visible spectrum, while extensively used, have been limited by the need for dark adaption to reduce the sunlight effect on the measurement. HSI was highlighted by the study as being the most objective of the three techniques but is limited by the complexity of the information it gathers and classifies. In Table 4, the three techniques are evaluated by the authors of [59]. The study concluded that the combination of thermography and hyperspectral sensors, or chlorophyll and hyperspectral sensors, improved the accuracy of the system to 89% from 78%.

### 4.1. Multispectral Imaging

**Figure 6:**
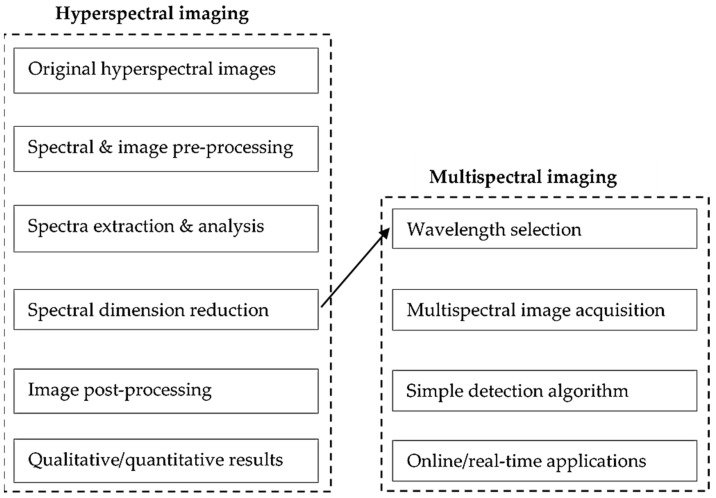
Procedure of hyperspectral and multispectral image analysis. Adapted from [60].

Multispectral imagery (MSI) uses sensors that measure reflectance across a broad band of the electromagnetic spectrum. The broad band from MSI sensors is composed of 3–10 distinct band measurements in each pixel from the images the sensor gathers [48]. In contrast, hyperspectral sensors measure reflectance in multiple narrower bands. MSI is more suited to providing a spatial pattern of diseased areas, while HSI can make line spectrum analysis. In Table 5, a sample review of previous research shows the variation of MSI on different plants and diseases, with the data analysis conducted across a multitude of methods. MSI has a much lower data set than HSI because the wavelengths of interest are predetermined [60]. The algorithmic difference between the two techniques is displayed in Figure 6.

### 4.2. Hyperspectral Imaging

Hyperspectral sensors (also called imaging spectrometers) are the next generation in spectral imaging, surpassing MSI radiometers prevalent in notable systems like LANDSAT, SPOT and IKONOS. These MSI examples are often used as the baseline to determine the accuracy of a HSI system. The information drawn from HSI is referred to a hyperspectral image data cube.

The HSI sensor continuously collects multiple images across numerous wavelength bands such that the spatial location (the X and Y coordinates correspond to the ground dimensions), which is represented by the pixel resolution of the sensor, compiles the data of all the measured wavelengths (the Z axis) [16]. HSI is a reflectance-based technique that offers more continuous coverage of the spectrum range than MSI (typically between 350–2500 nm) and can have a spectral resolution of <1 nm [11].

In the HSI model, different surfaces, such as the tree canopy or food crops, are represented by first- and second-order spectral statistics. The first- and second-order statistics of the spectral reflectance of each surface type can then be transformed in the spectral image process. In this process, the mean vector and covariance matrix of each surface reflectance type or class are used to evaluate the system’s performance. The HSI model is composed of the image condition module, the sensor module and the processing module.

**Figure 7:**
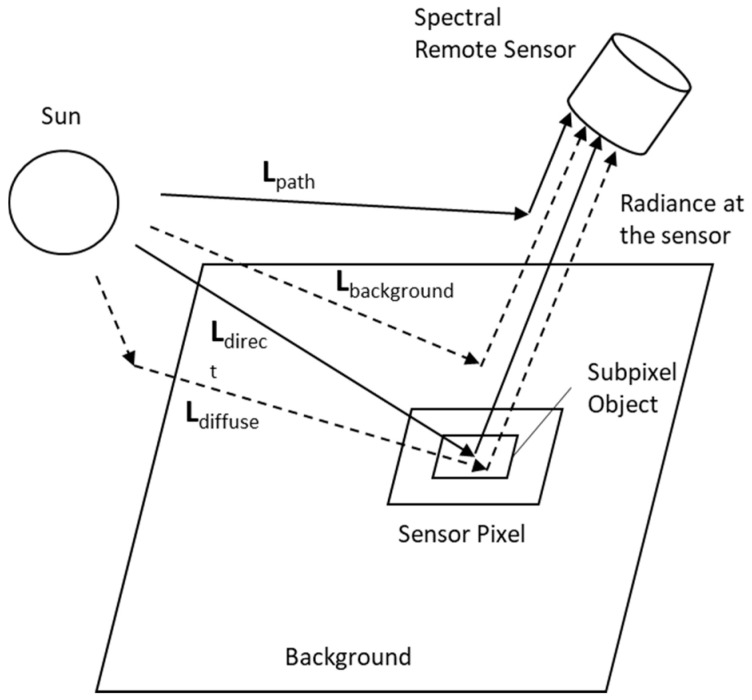
Radiance at the sensor from target reflection adapted from [75].

The image condition module describes the radiation transfer in the atmosphere as this accounts for the absorption, reflection and scattering that will affect the HSI performance. As a result of these influences, the radiance at the sensor, $L_{\lambda }$, is composed of three main elements: Direct reflection; path radiation, $L_{\lambda ,\;path}$; and radiation reflected outside of the target, as shown in Figure 7 [70].

(2) $$L_{\lambda }=L_{\lambda ,s}\cdot X+L_{\lambda ,\;path}+\left(L_{\lambda ,1}-L_{\lambda ,path}\right)\cdot X_{e}$$

where $L_{\lambda ,s}$ is the radiation at the surface, *X* is the vector of the surface reflectance, $X_{e}$ is the vector from the background and the final term, $\left(L_{\lambda ,1}-L_{\lambda ,path}\right)\cdot X_{e}$, eliminates the direct reflection. The mean, $\bar{L_{\lambda }}$, and the covariance, $\underset{L_{\lambda }}{\sum }$, can subsequently be calculated.

In the sensor module, the incident radiation passes through a detector, is amplified and is then evaluated for all of the spectral and spatial characteristics. The sensor module contains a spectral model, a spatial model and an error model.

The mean and covariance of the spatial model is related to the reduction in radiation and image degradation based on the coefficient of radiation reduction, *A*, the number of bands, *σ*, and the weight matrix, $W_{s}$.

(3) $$\bar{L_{L_{\lambda }}^{s}}=A\cdot \bar{L_{\lambda }}$$

(4) $$\sum_{L_{\lambda }}^{s}=\left[W_{s}\sigma_{A}\right]$$

The spectral model contains the centre wavelength as well as the adjacent wavelengths radiation [70]. The effect of these adjacent wavelengths needs to be accounted for in the mean, $\bar{S}$, and the covariance, $\underset{S}{\sum }$, where *B* is a linear transformation matrix of the spectral response functions.

(5) $$\bar{S}=B\cdot A\cdot \bar{L_{\lambda }}$$

(6) $$\underset{S}{\sum }=BW_{s}A\sum_{L_{\lambda }}A^{T}B^{T}$$

The error model consists of noise sources such as quantification error; dark-current noise, $n_{dark}$; radiometric error, $e_{rad}$; read-out noise and photon noise, as shown as a mean and covariance.

(7) $$\bar{Y}=\left(1+e_{rad}\right)\bar{S}+n_{dark}$$

(8) $$\sum_{Y}=\left(1+e_{rad}\right){\;}^{2}B\cdot W_{s}A\sum_{L_{\lambda }}A^{T}B^{T}+\wedge_{dark}+\wedge_{pho}+\wedge_{read}+\wedge_{quant}$$

These mean and covariance statistics for the three main models make up the spectral signal statistics from which the data processing can extract useful data.

The performance metrics of the HSI system is made up of three algorithms. One compares the spectral characterization accuracy based on the mean difference between the starting known reflectance and the received reflectance of the target. The second compares the probabilities that the sensor makes a detection opposed to a false detection. The third calculates the total error based on the total probabilities of a false alarm or missed detection [70].

In both MSI and HSI, the images are improved by lowering the unwanted intensity and improving the definitions of object boundaries by means of nonlinear diffusion for greater classification accuracy [71]. The nonlinear diffusion equation smooths the image by diffusing the noisy original intensity inside the image structures and reducing the variability of the intensity in the structure while retaining the areas of the high-intensity gradient at the edges of the structure [71].

(9) $$\frac{\partial u\left(x,t\right)}{\partial t}=\nabla \cdot (g\left(\nabla u\left(x,t\right)\right)\nabla u\left(x,t\right)$$

Here, the resultant smooth image is u(x,t), with the spatial coordinates x=(x,y) at a scale of *t*, and *g* is the nonlinear diffusion coefficient, the goal of which is to measure the dissimilarity between image gradients of two vector weighted pixels [71]. The hyperspectral image is described as a matrix, *V*, consisting of vectors, *v_i_*, related to the spectral signature *i*th pixel where *xy* is the total number of pixels [71].

(10) $$V={\left[v_{1},\;v_{2}\ldots v_{xy}\right]}^{T}$$

The image gradient, which uses a semi-explicit scheme that improves the smoothness and functionality, can then be described accordingly [71].

(11) $$\left(I-\;\mu G^{n}\right)V^{n+1}=V^{n}$$

(12) $$\mu =\;\frac{\Delta t}{\Delta x\Delta y}$$

where *I* is an identity matrix and *G* is a function of diffusion coefficients, normalized by the number of spectral bands, *m_z_*.

(13) $$g_{k+1}^{n}=g\left(\frac{\left|\left|v_{k+1}^{n}-v_{k}^{n}\right|\right|}{m_{z}}\right)$$

The key difference between HSI and MSI is that HSI can scan each pixel in broad-ranging wavelengths, encompassing more spectrum bands. This overcomes some of the inadequacies of MSI which are observed in accurately detecting plant biochemical properties and identifying optimal spectral ranges [20,48,57,72]. Identification of the optimal spectral ranges by HSI maximises the data collection of more relevant data. Research has demonstrated the benefits of HSI in improving accuracy, see (CC BY 4.0).

Table 6, the nature of the technique and the desired information means that all of the bandwidth spectral information is unnecessary [11,57,73,74].

**Figure 8:**
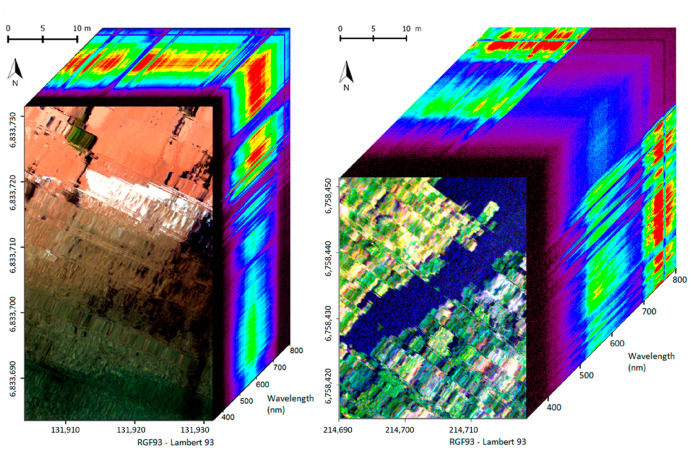
Representative spectral and spatial distribution of a hyperspectral image data cube of waterways [82]. (CC BY 4.0).

Like MSI, HSI benefits from rapid image data processing which crucially includes spatial information, making it more suitable to large-scale agriculture applications while still providing specific and accurate canopy-level information [77,78,79,80]. In each pixel, the reflectance spectrum is collected by HSI, as depicted in Figure 8, and within each image, the intensity of the reflected wavelength can be examined depending on the pixel quality. The spatial and spectral resolution influences the accuracy of the HSI system, with commercial CCD sensors available with >14 megapixels. For example, example a 1.3-megapixel camera with a 17 mm lens, 45 cm above a leaf, can make a data cube of 89.1 × 89.8 μm/pixel in the X and Y dimensions, with airborne sensor demonstrating a 1 × 1 m spatial resolution from a 1000 m altitude [81].

The data accumulated by HSI is quite extensive and, as discussed, excessive in comparison to MSI. The nature of HSI demands a high spectral resolution to detect small spectral deviations, allowing better analysis. However, due to the desired level of precision, the HSI technique is susceptible to spectral autocorrelation, where neighbouring narrow wavebands cause overlap and redundancy in the data [57]. To unpack the highly dimensional HSI data, data mining techniques are utilized to carry out the spectra analysis automatically and resolve the redundancy issue. The different types of techniques used are listed in Table 7. This approach allows plant genotypes to be characterized and the stages of the disease to be automatically identified and displayed, and displays the spectral deviations over time, which is useful for tracking the growth cycle of crops. The image processing involved in HSI is comprised of file reduction and sub setting, usually through principal component analysis (PCA), then defining the spectral library, the known data set which the data will use to then classify. There are many HSI image classifiers that use AI algorithms as well as vegetative indices for plant disease classification [81].

As depicted in Table 6, several studies have criticized the functionality limitations of MSI sensors in capturing surface properties both in spatial and spectral aspects [16]. By gathering data of adjoining narrow bands, HSI sensors can discern finer features that may be overlooked by the broad-band approach of MSI [16]. As a result, there is an increased classification accuracy in the use of HSI in comparison to MSI [83,84,85]. Because the HSI technique captures all of the wavelengths at each point, wavelength band selection can occur in post-processing, instead of narrowing the band selection before data collection as in MSI. This is especially advantageous for researchers and where gaps in the literature exist. In comparison to fluorescence-based models, HSI demonstrated a disease detection error of 11% relative to the 16% achieved by fluorescence-based models. A combined approach further reduced that error to only 6% [86].

HSI is disadvantaged by high cost, system fragility and complexity [16,69,87]. The restriction of the monitoring to narrow bands is to be further investigated since, depending on the scope of the application, wide-band measurements are more expensive, and the large data volume creates data overlap and strain on the system [11]. The literature is definitive in relating optimal spectral ranges to crop stresses, and the entire spectrum is not needed provided the application is sufficiently defined. Excessive data also impact the system in terms of data storage capacity, are computationally expensive and saturate radio datalinks [17]. Excessive data are somewhat offset by pre-processing feature extraction techniques. These feature extraction methods typically comprise independent component analysis, discrete wavelet transform (DWT), PCA, kernel PCA, derivative analysis, locally linear embedding, lambda by lambda correlation plots, partial least squares, vegetation indices and isometric feature mapping, as briefly described in Table 2 in the classification indices section and broadly in application in Table 7 [37,87]. These techniques reduce the dimensionality of the data and data redundancy and extract the useful data without identifying the redundant wavelengths. Using *R*^2^ values, the 15–20 hyperspectral narrowband (HNB) can achieve the same classification accuracy of the 240 HNB [88]. The optimal HNBs are selected based on the corresponding physical significance in vegetation. This approach is necessary when considering Hughes’ Phenomenon, in which each of the HNBs require training samples to ensure confidence in the classification [88]. Researchers need to balance the need for more HNBs, and therefore large training data, by removing overlapping HNBs within the system.

A review of the application of neural networks to HSI data was conducted, demonstrating the accuracy in disease detection by HSI [49]. The removal of the approximately 88% redundant data from HSI by data mining was performed by the authors of [37] to reduce data redundancy and dimensionality without eliminating unique data. HSI is relatively expensive due to its composition of narrow band filters, very sensitive detectors, spectrometers and 2D sensor arrays [16].

In disease detection, HSI is clearly advantageous in being able to acquire large amounts of data from which a lot of useful information can be extracted. However, there is limited maturity in the image processing systems, which limits feature extraction. Hence, few good automated systems are found in the literature [81]. Additionally, the HSI is still relatively expensive and requires substantial expertise to utilize, and the application to assess plant disease severity has not been fully explored and lacks adequate methods to process multiple diseases [81].

### 4.3. Thermography

Thermography is a passive technique in plant disease detection which exploits surface temperature measurements of leaves/crops/canopies to identify deviations indicative of abiotic or biotic stress. Thermographic cameras target the thermal infrared spectra, converting the pixels into temperature values, and the relative condition is then related to the leaf transpiration and water content [11]. Thermography is suitable for use in proximal and remote sensors and for pre-symptomatic diagnosis [100,101]. At a canopy level, thermography techniques are useful in wet conditions and in the detection of diseases that occur heterogeneously. However, the application of thermography is vulnerable to environmental conditions and lacks precision. Diagnosis of plant diseases from thermographic measurements of leaf transpiration is not very clear, although the implementation of sensor fusion is suggested as a future development [102]. Additionally, research has highlighted the suitability of infrared thermography in the detection of water stress across different genotypes, such as maize, soybean and cotton [103,104].

### 4.4. LIDAR

Active measurement systems like LIDAR are suggested as an alternative to the shortcomings of passive systems [105,106], as the latter are limited by the availability of ideal ambient lighting, impact of atmospheric conditions, changes in illumination and viewing angles and canopy structure, among other factors. Laser-based techniques mostly avoid problems associated with light, allowing for night measurements. One of the key benefits of LIDAR is its ability to take measurements during the day and night, a driving factor for its implementation in NASA’s Active Sensing of CO_2_ Emissions over Nights, Days and Seasons (ASCENDS) mission. In this case, Differential Absorption LIDAR (DIAL) made it possible to the gap in knowledge of oceanic carbon sinks at night [107]. As LIDAR measures range, height errors are reduced while spatial reflectance properties and leaf biochemistry can be obtained. In fact, several sources of bias are removed due to this interaction of the surface and atmosphere [107]. Additionally, with a small laser footprint, LIDAR measurements can be taken between gaps in thick clouds. LIDAR systems in airborne platforms offer greater flexibility in capturing data; have a high rate of data delivery, increase elevation accuracy, particularly in difficult terrain; and are capable of a high level of automation [108].

Practical difficulties of in-field implementations such as complex canopy structures, colour variations, textures, lighting conditions, shadows and heavy post-processing costs are key limitations of VIS-based crop monitoring systems, which have led to the development of a dual LIDAR UGV-based system acting in a pseudo stereo vision as an alternative low-cost, high-accuracy system [109]. Hyperspectral data combined with LIDAR shape profiling on a UAV platform is utilized to quantify the photosynthetic processes in forest vegetation [110]. Using data fusion, the 3D LIDAR point cloud data and hyperspectral reflectance data are combined to predict the biochemical traits present at the canopy level that are highly correlated. Despite difficulties in the vertical estimation of biochemical traits, research has concluded that the fusion of spectral imagery and LIDAR has a wide set of applications, including biochemical traits, tree species mapping and biomass estimation [110]. This is undercut by the difficulty in combining the two sets of disparate data, only achieved with significant advanced image processing. Using DIAL technology in an airborne platform, local biomass and carbon levels can be estimated. By integrating a LIDAR plant height detecting sensor with a passive optical NDVI sensor, the system can better estimate biomass and explore complex in-field relationships in tall fescue [111]. Unlike the carbon dioxide wavelength approach described, these methods typically revolve around using LIDAR volumetric measurements and canopy height measurements to determine variations.

#### Carbon Dioxide Absorption Spectroscopy

Using DIAL, it is possible to detect nonvisible symptoms of agricultural diseases based on the CO_2_ concentration over the canopy. Ambient CO_2_ over canopies does not change in the early morning or at night, whereas it decreases significantly at midday (about 12 pm) with a changeable amount of 411−2.5 = 408.5 ppm [112]. Therefore, it is important to detect the changeable amount by sensors because it is an indication of CO_2_ absorption from plants. The amount of absorbed CO_2_ is maximized by healthy plants. However, the amount of absorbed CO_2_ is reduced by stressed plants. Sensors are required to measure these small changes. Stressed plants are caused by a lack of water, excess of fertilisers, increased salt in the root zone and diseases. In addition, CO_2_ absorption consumed by plants is necessary to generate food and energy for their growth and cellular respiration. In field conditions, CO_2_ concentration over or in the canopy is extremely dynamic due to the diffusion and turbulence processes.

Carbon dioxide sensors for the agricultural sector have a few distinctions among the conventional industrial sensors regarding the determination of environmental parameters. The sensors are essential for performance under extraordinary conditions of temperatures, pressure and humidity. The agricultural environment is difficult to completely define, with numerous microorganisms and other biological species impacting the parameters under study [113]. The agricultural sensors need to be capable of handling highly variable processing and of supporting users in interpreting the calculation data as clearly as possible. The accuracy of monitoring CO_2_ is becoming significant in various agriculture applications, e.g., CO_2_ soil respiration, gas exchange, observing atmospheric gas, in the alcohol and beverage industry [114], the scanning of biogas composition [115] and the identification of freeze damage in orange fruits [116]. The sensitivity of CO_2_ concentration detection is a powerful invention which we can apply to measure the anomalies of CO_2_ concentration associated with stressed plants. In Table 8, the fluorescence-based model is compared with the how LIDAR measurements of CO_2_ absorption are used to discern plant health.

Variations of CO_2_ concentration in fields are extremely complex based on the factors of soil respiration, plants photosynthesis and air turbulence. The CO_2_ concentration changes of soil and plants are much easier to detect than air turbulence changes. However, LIDAR techniques can solve air turbulence problems with a remarkably fast measurement process.

### 4.5. LIDAR Shape Profiling and 3D Scanners

**Figure 9:**
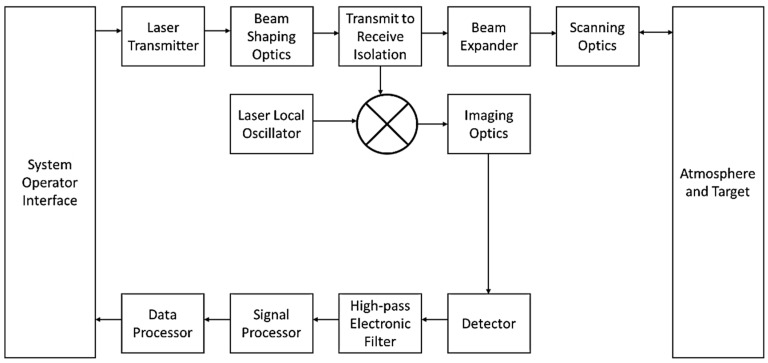
Light detection and ranging (LIDAR) profiler block diagram. Reproduced with permission from Elsevier [108].

A typical LIDAR shape profiling system (also commonly known as a 360 scanner) architecture is shown in Figure 9, identifying some of the key components of the system.

The LIDAR sensor is used to measure the relative position of an object in five dimensions, which are the x, y (ground dimensions) and z (height above canopy) coordinates and the time and the intensity of the reflected light by which laser source emits light and the detector receives the reflected light [117]. In fact, the distance from the laser source to the target is calculated by two types of techniques: The time of flight method for the pulse-based light source and phase modulation method for continuous wave light. These two methods are distinctive in that multiple range measurements from a single pulse are recorded by pulse-based methods, while a single range measurement is offered by continuous wave methods [118]. The spatial information has been employed to enhance comprehensive forestry applications. However, little research has been conducted on the intensity of the reflected light due to problems calibrating intensity [119,120]. Optical remote sensing methods have widely applied reflected information in many applications, whereas the potential of intensity information from laser scanners has little been studied. Multispectral laser scanners give a comprehensive array of wavelengths for current technical enhancements [117,121,122].

Several vegetation applications primarily based in laboratory conditions have been improved using multispectral laser systems. The dual-wavelength Spectral Ratio Biospheric LIDAR, which indicates the modifications of ratio between the red and near-infrared wavelength regions from a phenology cycle of a tree canopy, was studied by Rall and Knox [123]. The Multi-wavelength Airborne Polarimetric LIDAR, which utilises a dual wavelength (532 nm and 1064 nm), can differentiate reflected spectrum among tree species [124]. Others have proposed the Multispectral Canopy LIDAR approach, designed as a prototype of the airborne sensor which employs a single tuneable laser to allow the measurement of NDVI and PRI [125]. A multispectral LIDAR system was developed, based on a laboratory prototype, to measure at four wavelengths (556 nm, 670 nm, 700 nm and 780 nm) to detect nitrogen stresses on rice leaves by modifying the optical properties and reflected spectrum [126]. However, excluding systems that include a scanning mechanism, all of the systems have not been obviously operated in the field, which limits their instant value and feasibility for in situ calculations of vegetation canopies [126]. The capacity of laser reflectance ratios (in the range of 9–11 µm wavelength) has been illustrated in research in order to distinguish healthy and stress plants. However, particular biochemical properties and different types of stress were not distinguished by the relationships between these ratios [127]. Leaf biochemical properties are potentially measured by a multispectral laser scanner, although the study was carried out with a tiny number of samples [128]. The accuracy of LIDAR measurements is limited to shorter ranges in comparison to imaging sensors. Some factors, i.e., remarkable fluctuations of laser energy on the focal plane and some nonlinear propagation influences (bleaching and thermal blooming), are caused by atmosphere turbulence, which also generates serious attenuations of laser beams propagating in the atmosphere [129,130,131].

The integrated path differential absorption (IPDA) LIDAR technique transmits (at least) two laser pulses with similar wavelengths, as demonstrated by NASA’s space-based ASCENDS mission platform to detect CO_2_ and O_2_ concentrations. The approach allows for greater flexibility, is less wavelength-dependent, removes the scattering effects of thin clouds and surface scattering and is also used in conjunction with the DIAL technique [107].

### 4.6. Bistatic LIDAR System Concept

LIDAR absorption spectroscopy systems have been proposed as a feasible method to remotely sense the atmospheric components as the laser beam at the detector can discern the characteristics of the medium the beam passed through [105]. The use of the DIAL measurement principle takes advantage of the wavelength-selective absorption properties of molecular species. LIDAR can be classified as monostatic or bistatic based on the use of a single aperture (monostatic) or separate apertures (bistatic) to transmit and receive. Most common DIAL systems are monostatic (single-pulsed), but these require complex and expensive components to detect the very weak backscatter by aerosol particles [132]. As the bistatic LIDAR layout reduces cost, size, weight and power compared to monostatic LIDAR one, a bistatic LIDAR system has been proposed as a cost-effective solution that can be integrated on small drones for the early detection of crop diseases [132,133]. The extension of this method to the agricultural sector was evaluated to measure CO_2_ concentrations associated with photosynthesis and respiration [105,134].

The bistatic LIDAR system consists of a transmitter that emits a continuous wave or pulsed laser beam of precisely known power characteristics. The amount of laser energy that arrives from the transmitter, which is less than the emitted LIDAR radiation because atmospheric molecular/aerosol concentrations absorb or scatter the radiation (as shown in Figure 9), is measured by the receiver. Generally, the power of a transmitted laser beam is attenuated and scattered by the atmosphere before it is received by the detector. Rayleigh, Mie and nonselective scattering are the primary wavelength attenuation types. Direct and scattered light from the sun or other sources also affects the receiver measurement.

Two individual wavelengths are used in the DIAL technique. They are divided in the “on” and “off” absorption lines. The on-absorption line is chosen corresponding to a major vibrational band of the targeted molecular species. The off-absorption line is selected close to the first wavelength but away from the vibrational band of the targeted molecular species so that the difference in cross-sections $\Delta \psi ≜\psi \left(\lambda_{ON}\right)-\psi \left(\lambda_{OFF}\right)$ is maximised [135]. Applying the DIAL principle allows these parasite factors to be neglected as they affect both wavelengths used by the system. This relationship of attenuation effects of different aerosol and molecular species is typically calculated by Beer–Lambert’s law as given by:

(14) $$\tau_{\lambda }=\frac{P_{RX}}{P_{TX}}=e^{-\gamma \cdot z}$$

where the transmittance, $\tau_{\lambda }$, is characterized by the ratio of received power, $P_{RX}$, to transmitted power, $P_{TX}$. Here, γ is the scattering coefficient and *z* is the path length.

(15) $$\gamma \left(\lambda \right)=\alpha_{m}+\beta_{m}+\alpha_{a}+\beta_{a}$$

The scattering coefficient is made up of α(λ), a molecular $\alpha_{m}$ and aerosol absorption coefficient, $\alpha_{a}$; and scattering coefficient, β(λ), a molecular, $\beta_{m}$ and aerosol scattering coefficient, $\beta_{a}$, at a selected wavelength. The scattering caused by visible light ($\delta_{VIS}$) can be neglected based on Sabatini et al. [108]. Molecular scattering (*β_m_*), aerosol absorption (*α_a_*) and aerosol scattering (*β_a_*) are also ignored due to DIAL measurement principle. The molecular absorption ($\alpha_{m}$) is calculated as followed:

(16) $$\alpha_{m}\left(\lambda ,l\right)=\;\sum^{\;}\overset{\lambda_{2}}{\underset{\lambda_{1}\;}{\int }}\sigma_{i}\left(\lambda ,P,\Theta \right)\cdot d\lambda \cdot \ddot{\left[N_{i}\right]}$$

where $\lambda_{1}=\;CWL-\frac{FWHM}{2}$ [nm]; $\lambda_{2}=\;CWL+\frac{FWHM}{2}$ [nm]; CWL = center wavelength of the laser diode [nm]; FWHM = full-width half maximum of the laser diode band width [nm]; $\sigma_{i}\left(\lambda ,P,\Theta \right)$ = absorption cross-section of *i*th molecular gases along the laser beam as a function of wavelength (λ) in nm, pressure (P) in atm and temperature (Θ) in *K* based on HITRAN2016 database $\left[cm^{2}\cdot mol^{-1}\right]$; $\ddot{\left[N_{i}\right]}$ = molecular volume concentration of *i*th gases $\left[\mathrm{mol}\cdot cm^{-3}\right]$; $\alpha_{m}\left(\lambda ,l\right)$ = total absorption coefficients of molecular gases as a function of wavelength (λ) in nm and the baseline of transmitted beam (*l*) in cm $\left[{\mathrm{cm}}^{-1}\right]$. The connection between photodetector energy for the master wavelength, P_Rx_(λ_ON_), and for the non-master wavelength, P_Rx_(λ_OFF_), in a direct path can be calculated as [131]:

(17) $$R_{ON/OFF}=\frac{P_{\mathrm{Rx}}\left(\lambda_{\mathrm{ON}}\right)}{P_{\mathrm{Rx}}\left(\lambda_{\mathrm{OFF}}\right)}=\frac{\tau_{ON}}{\tau_{OFF}}=exp\left\{-\left[\psi_{acs}\left(\lambda_{ON}\right)-\psi_{acs}\left(\lambda_{OFF}\right)\right]\int_{0}^{l}\ddot{\left[N_{CO_{2}}\right]}\left(l\right)dl\right\}$$

Using the scattering coefficient in Equation (3), the net CO_2_ concentration ($\ddot{\left[N_{CO_{2}}\right]}$) can be determined by the following equation:

(18) $$\ddot{\left[N_{CO_{2}}\right]}=\frac{ln\left[P_{Rx}\left(\lambda_{OFF}\right)/P_{Rx}\left(\lambda_{ON}\right)\right]}{l\left[\psi_{acs}\left(\lambda_{ON}\right)-\psi_{acs}\left(\lambda_{OFF}\right)\right]}$$

The DIAL measurements mainly focus on molecules vibrational absorption associated with laser wavelengths. This signal analysis enables an estimate of the CO_2_ concentration. Various DIAL systems can be integrated with RS systems due to the emergence of lightweight, powerful laser sources and systems. Active monostatic configurations are employed with DIAL configurations for atmosphere sounding that measures not only the elastic backscatter produced along the external path but also the radiance of the illuminated Earth surface in the direction of the airborne system. In addition, numerous DIAL systems have been improved to measure the concentration/column density of different significant molecule species based on the development of powerful tuneable lasers [131,136,137,138,139,140,141,142,143,144,145,146,147,148,149,150,151,152].

#### LIDAR Laser Beam Propagation in the Atmosphere

The performance of LIDAR is underpinned by the attenuation of the laser beam through the atmosphere based on the characteristics of the atmosphere and the power and wavelength of the beam. At low output powers, these propagation behaviours are linear, while at sufficiently high output powers, the behaviours are nonlinear [153]. The linear behaviours include absorption, scattering and atmospheric turbulence. The nonlinear effects include thermal blooming, beam trapping, bleaching and atmospheric breakdown [153].

Molecular line absorption is a dominant cause of attenuation dependence on the laser wavelength. In this case, the laser encounters one (or several) molecular species at the specified wavelength as it propagates through the air. The energy from the laser is then attenuated by the molecular species in its path. Knowing how the wavelength corresponds to a certain molecular species (high-resolution data is available in programs such as HITRAN), the attenuated energy can be calculated and used to determine concentrations of various molecular species present in the medium.

Continuum absorption describes a type of molecular absorption as a result of molecular clusters. A part of this type of attenuation is atmospheric scattering, where a directional change leads to a reduction in the beam intensity, especially over large paths. There are different types of scattering, which are characterized by the physical size of the scatter. In small air molecules, attenuation leads to Rayleigh scattering, and for aerosol-sized molecules, it leads to Mie scattering. For even larger-scale molecules, the scattering can be described by diffraction.

Rayleigh scattering often has a much larger effect on the propagation than molecular absorption in the VIS and NIR and is characterized by the scattering cross section in the equation below [153].

(19) $$\sigma_{s}=\frac{{\left(\frac{e^{2}}{m}\right)}^{2}\omega^{4}}{6\varepsilon_{0}^{2}\pi c^{4}\left[{\left(\omega_{0}^{2}-\omega^{2}\right)}^{2}+{\left(\Gamma \omega \right)}^{2}\right]}$$

which characterizes the scattering of a single dipole radiator by the electron charge, *e*, the natural frequency, *w*_0_, and a damping coefficient. Mie scattering considers the case where the wavelength of the laser beam is scattered by particles of similar sizes. The size, dielectric constant, shape and absorptivity of the particle is accounted for in Mie scattering. To calculate the atmospheric attenuation coefficient, it is important that the quantities of all the different sizes of aerosol particles are known, which can be difficult. Mie scattering is dependent on atmospheric conditions such as humidity [153].

(20) $$-\frac{dI}{I}=\frac{K\pi a^{2}NAdz}{A}=N\sigma \left(a,\lambda \right)dz$$

which describes the decrease in beam intensity as a factor of the attenuation factor, *K*, and the Mie attenuation coefficient. Propagation through the effects of precipitation is significantly attenuated and is dependent on the specific atmospheric condition, which the authors of [154] explored.

The beam is also subjected to propagation through atmospheric turbulence where turbulence energy is introduced in large scales. Turbulence is inherently nonhomogeneous and non-isotropic, making it difficult to model with dynamic vertical profiles dependent on weather factors such as wind speed, pressure, temperature and humidity. In describing beam attenuation from turbulence, the refractive index structure coefficient is considered as the most important parameter [153]. The coefficient uses the pressure and temperature difference at two points as described below [155].

(21) $$C_{n}=\left[79.10^{-6}\frac{p}{T}\right]C_{T}$$

(22) $$C_{T}=\sqrt{{\left(T_{1}+T_{2}\right)}^{2}}\frac{1}{\sqrt[{3}]{r}}$$

**Figure 10:**
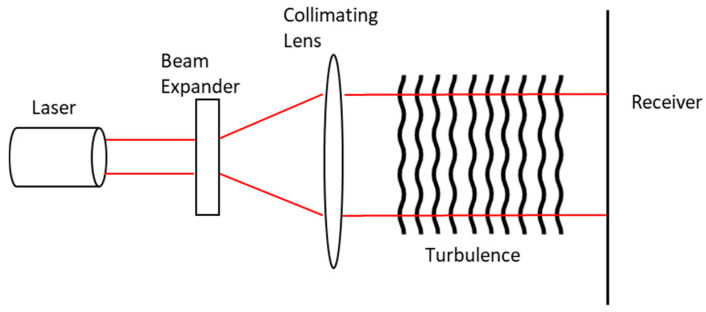
Laser beam attenuation through atmospheric turbulence. Adapted from [153].

The turbulence effects on the laser beam, illustrated in Figure 10, are affected by the transmitted beam properties, the refractive index structure coefficient and the inner and outer scale of the turbulence. The turbulence can cause ‘beam wander,’ where the laser beam is deflected by turbulence cells that are larger than the beam diameter In this case, the beam diameter remains the same, but the beam is deflected from its path. When the turbulence cells are smaller than the beam diameter, the diffraction and refraction occurs, distorting the beam intensity profile. Both cases can occur simultaneously [153].

These types of attenuation are linear as the laser beam does not affect the air it passes through. However, for beams with high irradiance, attenuation induces thermal changes in the air it passes through, resulting in nonlinear effects as the temperature affects the density and index of refraction, in turn altering the beam’s irradiance distribution [153]. These types of effects include thermal blooming, where the temperature at the centre of the beam rises, causing an expansion resulting in a defocusing of the beam [153].

The system performance at peak irradiance for nonlinear effects such as atmospheric turbulence, random jitter, thermal blooming and diffraction is given in terms of the output power, *P*, the attenuation coefficient, γ, and the contributions of diffraction, jitter and turbulence given by $\left(a_{d}^{2}+a_{j}^{2}+a_{t}^{2}\right)$.

(23) $$I_{P}=\frac{Pe^{-\gamma z}}{\pi \left(a_{d}^{2}+a_{j}^{2}+a_{t}^{2}\right)}$$

Additionally, aerodynamic effects can produce optical anomality’s as a result of the viscous flow of laminar and turbulent boundary layers, as well as the inviscid flow. In the mid-IR, turbulent surface boundary layers are likely to cause optical aberrations for high-powered lasers. In this case, increases in the Mach number and Reynolds number result in more complex interactions and optical aberrations for airborne laser systems [131].

### 4.7. Hyperspectral and LIDAR RS Fusion

As discussed, the canopy scale introduces variables that degrade sensors’ ability to distinguish important relationship between the reflectance spectrum and plant health. The combined use of passive HSI and active LIDAR has demonstrated several benefits, enabling the system to filter out peripheral spectral information in the canopy. The analysis of pigmentation was shown to improve above the canopy with the addition of LIDAR [156] by utilizing LAI canopy measurements derived from the LIDAR sensor to extrapolate HSI sensor data of the foliar chlorophyll concentrations and to quantify the whole canopy chlorophyll concentration [57,157,158]. The ability of LIDAR to give complementary data on leaf biochemistry and vegetation type to passive systems is significant in plant disease detection [106]. The development of hyperspectral LIDAR systems is an expanding area of research as illustrated by the consistent integration and testing shown in the research [29].

The trends shown in research show that the identification of molecular gases in LIDAR applications is of research interest for both HSI–LIDAR data fusion in agriculture applications. The most recent developments have used the laser propagation principles in Ramen scattering to identify and quantify nitrogen and oxygen molecules as a means of determine atmospheric composition. Most applications of HSI–LIDAR data fusion focus on LIDAR 3D scanning technology as means of calculating tree height for biomass estimation. This approach underutilizes the data that could be retrieved by the LIDAR sensor, which can perform similar plant health analysis as HSI, through an understanding of the laser propagation properties of LIDAR. This approach makes the LIDAR sensor much more versatile in a precision agriculture application, and combining the LIDAR sensor with the HSI technique would mitigate some of the shortcomings of the HSI, such as variations in the canopy that BRDF techniques have to be used to compensate for. Further research into in-field LIDAR-based chemical detection is required to fully exploit any potential data fusion, but there are clear benefits of increased accuracy by taking the reflectance based (HSI) and molecular absorption based (LIDAR) combination in the context of challenging environments in agriculture applications.

## 5. Food Quality Analysis

### 5.1. Fluorescence Spectroscopy

Fluorescence spectroscopy was used to estimate the ripeness of mandarins [47,159]. Here, the fruit maturity was indicated by ratio of sugar content to acid content through fluorescence spectroscopy analysis of the peel. The ratio of sugar to acid content strongly influenced the taste, making it a much more desirable quality to analyse instead of size and firmness [159]. Research has also explored pigmentation and flavonoids in apples as an indicator of fruit quality with the utilization of a fluorescence sensor [23]. The study concluded that chlorophyll fluorescence was a good gauge of food quality related properties, flavanols, anthocyanins and chlorophyll in apples. The use of fluorescence spectroscopy was found to help monitor the ripening process and, more subjectively, to assess the colour quality (determined by the anthocyanin measurement) for market viability. Proximal sensing using fluorescence spectroscopy was used in monitoring grape maturity by the authors of [160,161]. Again, the anthocyanin measurement was emphasised for maturity. The advantages of the spectroscopy approach was highlighted through a more detailed maturity grading of peaches [162].

### 5.2. Multispectral Imaging

By incorporating MSI data obtained from a UAV with a handheld fluorescence spectrometer, researchers were able to assess grape maturity, acidity and sugar content [161]. A comparison of MSI with HSI in analysing the presence of canker disease on grapefruit finding significant advantages in MSI’s simpler image data analysis structure [60]. MSI was also used in the assessment of apple firmness [163] and of firmness and maturity in peaches [164]. Airborne MSI and thermal imaging was used to assess fruit water stress and quality of peaches, oranges and nectarines in relation to different irrigation regimes based on the PRI [165].

### 5.3. Hyperspectral Imaging

Beyond disease classification and quantification, HSI is increasingly used to assess food quality under constrained laboratory conditions (proximal sensing). In comparison to MSI, HSI is especially useful in identifying the optimal wavelengths [76,99] to correlate the reflectance change to a biotic change, as demonstrated with the detection of bruise spots, fungal and faecal contamination of apples by the authors of [99,166,167,168,169], as well as the identification of chilling injury in cucumbers [170], fungi in corn [171], and bruises in strawberries [172] and even in relation to the quality of chicken carcasses [77]. To truly take advantage of the HSI capabilities, it is essential to examine the available information in the spatial dimensions and use HSI more extensively in RS canopy-scale applications. Using a combination of HSI and LIDAR on a UGV platform, it was found that the maturity of mangoes could be assessed based on the detection of dry matter in peaches, greatly improving in situ measurements [173].

### 5.4. LIDAR

LIDAR sensor applications in fruit quality are currently focused on counting and tracking the fruit. A unmanned aerial vehicle (UAV)-based platform utilizing a multi-sensor approach of LIDAR, MSI, thermal imaging and navigational sensors was used to extract plant health data [174] including the plant morphology, canopy volume, NDVI, LAI and fruit counts. Multi-sensor data fusion approaches allowed researchers to rebuild the canopy dimensions and differentiate between the heights of different species of trees, a key indicator in plant phenotyping. MSI and LIDAR were also used to accurately identify plant stressors. Additionally, using a support vector machine classifier, the UAV platform could count and track the fruit of the cultivars. This helps predict the yield and informs the growers’ decision-making.

## 6. Remote Sensor Platforms

True exploitation of RS for precision agriculture invariably lies with the application to a canopy scale to fully appreciate the spatial information advantages, especially for large agriculture areas. Whereas the in situ and RS sensor technologies have been extensively tested in heavily controlled circumstances, in-field airborne applications are increasingly demonstrating their usefulness in time saving both in processing and for large areas, spatial information and accuracy. One potential drawback is the diminishing resolution of MSI, HSI and LIDAR sensors as the distance between the sensor and target area is increased. However, this can be actively managed as part of the flight mission planning. Gleaning the reflectance of the entire canopy also introduces factors such as the variation in leaf layers (LAI); the orientation of the leaves, called the leaf angle distribution (LAD); weather; shadows and non-leaf elements like soil [57]. These factors complicate the relationships between spectral reflectance and plant health.

**Figure 11:**
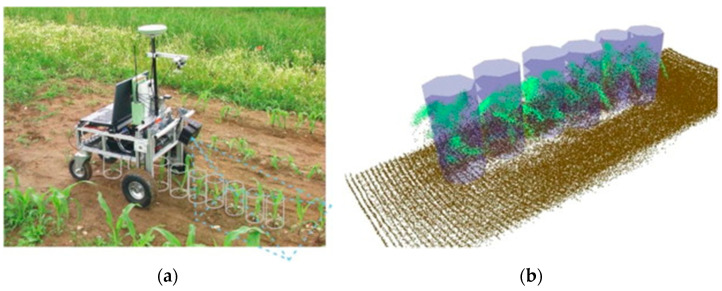
(**a**) Unmanned ground vehicles (UGV) platform and (**b**) 3D data points collected from LIDAR sensor. Reproduced with permission from Elsevier [179].

The prevalence of agricultural robots or unmanned ground vehicles (UGVs) is notable in applications such as seed identification, field scouting and harvesting [175,176,177]. Of interest, in the scope of this review, the in-field information can be used in health assessments. UGV platforms need to perform in complex, variable environments while being multipurpose and cost-effective to be viable. As a result, UGV platforms typically feature advanced sensors, including obstacle avoidance, high-accuracy navigation systems and 3D mapping systems. Computer vision-based or stereo-based cameras are commonly used for relative positioning and determining the crop row locations, building maps and collision avoidance [178], with the trend of advocating the use of 3D LIDAR sensors that better adapt to complex outdoor environments. Resolving this localization and navigation problem is central in the effectiveness of UGVs [179]. This approach was applied to maize plants but was limited by the resolution of the sensor, as seen in Figure 11. The approach was able to detect the plant and the ground to map their locations using the point cloud and use machine learning to identify different plant species.

Using a similar approach, utilizing 3D LIDAR in the mapping of forest areas on a UGV platform demonstrated the ability of UGV platforms in uneven, complex terrains while providing an efficient approach for the calculation of tree locations and diameters [180]. UGVs such as VINBOT are used for yield estimation, in this case, in vineyards, where the UGV excelled at carrying heavy sensors (>50 kg). As a result, the UGV design must have a low centre of gravity for platform stability but requires elevated sensors to acquire the relevant data [181].

UAV, aerial and satellite RS platforms for plant disease detection are continuously improving their spatial and temporal resolutions, thus increasing the number of suitable applications for precision agriculture. The distinction between platform suitability is heavily biased toward the mission specificity, spatial scale and cost. The advantages of each remote sensing platform are summarized in Table 9. Ground and handheld platforms are used for high spectral resolutions. Aerial and space platforms are more applied for spatial information at reduced spectral resolutions. Aerial platforms are also expensive but can be much more flexible than satellites [182]. Satellites offer greater worldwide coverage than aerial platforms but are limited by their orbit. Space-based platforms are suitable for large areas at the expense of resolution, as discussed in research, and are susceptible to clouds. Space-based platforms are expensive, and are also constrained by the orbit, which dictates when measurements can be taken [183]. This issue of the timing of measurements can be critical in some vegetation. NASA has demonstrated LIDAR CO_2_ column absorption capabilities on ground-based, aerial and space-based platforms [107]. Both aerial and space platforms can be severely hampered by weather and cloud coverage.

UAVs have been demonstrated to be competitive, with more well-tested imagery acquisition platforms such as aerial and satellite in large part due to low operational cost, high resolution and mission flexibility. The flexibility of UAV was highlighted for vineyard diseases that occur in small heterogenous areas, which are observable only for brief periods [69]. Detecting plant disease and monitoring food quality are of significant interest in vineyards. It is noted that, in heterogenous environments, in comparison to the UAV platform, imagery resolution using aerial and space-based platforms is low and provides an inaccurate representation of the field [183].

UAV-based platforms in precision agriculture have been used and validated for the detection of water stress [184], biomass estimation [185], measurement of the LAI [186] and detection and classification of plant diseases [187]. UAV-based visual sensors have shown high levels of accuracy in the disease detection of a variety of species [177]. A UAV with MSI high-resolution cameras was used to characterize the progression and severity of rice diseases through NDVI, showing its success as a quick and accurate disease detection and highlighting the flexibility of the UAV platform and advances in resolution enabling the method to monitor and assist in plant breeding [188]. Recently, a UAV-based platform was developed utilising multispectral and hyperspectral cameras for plant health detection [189]. The results were used in conjunction with UGV platforms to estimate yield and to isolate infected plants, with the potential for removal of weeds and unhealthy plants through a UGV robotic arm. Measuring the water stress is a common application for UAVs in precision agriculture, with the combined use of thermal, hyperspectral and fluorescence sensors being experimentally validated and used to improve irrigation practises [190]. There are a variety of different UAV platforms, including fixed wing, single rotor, multirotor and balloon, offering different capabilities and suitability to precision agriculture. There are advantages and disadvantages of each of the distinct UAV platforms in the implementation of MSI, HSI, thermal sensors and other active sensors [18]. Largely, the study concluded that integrating sensors to the UAV is needed to overcome weight, navigation and vibration considerations.

Plant disease detection techniques are driven by governing body regulations such as the European and Mediterranean Plant Protection Organisation (EPPO) to avoid crop yield losses. An example of this work demonstrates how MSI data from a UAV platform can be combined with proximal sensors to assess fruit quality [161]. The high spatial resolution of UAV imagery in the range of 1–10 cm accuracy [183,191], as well as the low operational cost and flexibility of the platform, have led to use in small farm applications [192]. A study also compared UAV, aerial and space-based platforms, using MSI to determine the NDVI of two Italian vineyards [183]. The study characterised the comparative RS platforms performance, as shown in Table 9 and Table 10. The UAV platform obviously suffered from range, endurance and payload performance in comparison to the others. However, the study extolled the flexibility, resistance to cloud cover, high resolution and precision of the UAV platform, particularly in relation to fragmented farm areas. The cost analysis performed by the same study found that UAVs were cost-effective in image acquisition for areas smaller than 5 hectares [183].

### UGV and UAV Cooperative Approaches

**Figure 12:**
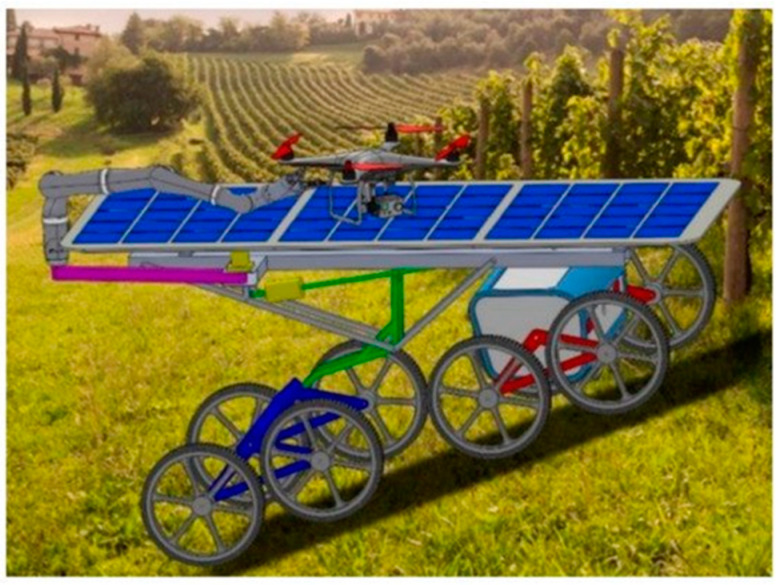
UGV-unmanned aerial vehicle (UAV) cooperative concept [176]. (CC BY 4.0).

UGVs are advantageous over other platforms in their ability to carry many sensors that can be heavier and bulkier than UAV applications which are used for high-resolution data acquisition. Conversely, the UGVs struggle in complex unstructured environments, are usually slow moving and are hindered by ground obstacles. The research suggests a trend toward cooperative UGV and UAV systems for improved coverage and resolution. In this model, the UGV performs complimentary data acquisition and acts as a mobile charger for the UAV, delivering it to deployment locations as seen in Figure 12 [189,193,194,195].

## 7. Data Analysis Methods

Adopting the right combination of sensors, platforms and data analytics in agriculture helps reducing the proportion of manual operations and leads to improved yields through the effectiveness and efficiency gains that only wide-area remote measurements can achieve. This ongoing evolution is increasingly driven by the application of AI to perform nonstandard tasks, which expand the range of activities that can be performed in precision agriculture to allow for data-driven decision-making from the operators. For instance, it is functionally essential for RS sensors such as HSI, MSI and LIDAR to utilize machine learning (ML) techniques to sort, segment and classify the large amount of data acquired. Correlating the spatially and temporally distributed data from one or more sensors to definitive classifications and diagnoses indicating plant health is achieved through a variety of different ML techniques, typically tailored to the specific crop, conditions, sensor and platform. ML in precision agriculture has expanded with increased sensor resolution and has been paired with modern algorithms to allow for multi-data fusion. AI techniques are used in two distinct ways in remote sensing platforms: For mission control (navigation, obstacle avoidance, etc.) and for processing the data from the sensors into interpretable information for the operators. The methods used to filter the raw spectral data and quantify it into statistical data from which disease classification can be inferred greatly influence the accuracy of the system and warrant further exploration. As observed in the literature (see Table 3, Table 5 and Table 7), numerous research studies have aimed to develop superior data analysis methods in plant health applications. These studies have prioritized the accuracy of the system over other considerations, such as cost, real-time analysis, suitability for in-field testing and suitability for a remote sensing platform. The following is a short overview of the most prominent data analysis techniques used in plant health monitoring sensors.

Future work in data analytic methods for precision agriculture should examine AI data fusion, and the use of digital twins and meta-reasoning as methods to improve the accuracy and efficiency of RS systems. The observed research clearly trends toward a multiple sensor data fusion approach in the health monitoring of crops. The use of appropriate techniques to collate the data from different sensors in order to improve the classification accuracies is of significant interest. As observed in the section concerning HSI–LIDAR (3D scanner) data fusion, the data collected does not really overlap. The HSI system measures the reflectance, which is used to discern information about the photosynthetic efficiency, and the LIDAR 3D scanner usually measures the heights of the crop to measure biomass. The combination of these results provides more information. However, both HSI and bistatic LIDAR are a measure of photosynthetic efficiency, and with their combination, there is opportunity to share information between the sensors to better improve each technique.

The application of meta-reasoning to the data analysis techniques is an emerging technique designed to measure the efficiency of the data analysis. Meta-reasoning examines and controls the resources and time that the data analysis takes for each task. This optimizes the decision-making and quality of the result.

### 7.1. Partial Least Squares Regression

Partial Least Squares Regression (PLSR) uses a linear multivariate model to relate and model the structure of two data matrices [196]. PLSR accuracy increases with the number of observations and variables, even when the data is noisy, collinear or incomplete. In this way, PLSR overcomes the problem of overfitting encountered with increasing observations [197]. The spectral PLSR method was shown to quickly and accurately predict the photosynthetic capacity. However, this accuracy is susceptible to variation in the genotypes of a single species of crop [198]. PLSR was used to determine the nitrogen and phosphorus content in barley plants using HSI of the canopy, with an accuracy of 81% for phosphorus, 74% for nitrogen and 75% for predicting the growth stage [98]. However, in cucumber leaves, the phosphorus content was not able to be accurately determined by PLSR methods with an in situ spectroradiometer due to a high nonlinearity correlation, resulting in the use of SVM and ANN [55]. In determining the plant biochemical properties, it was found that PLSR was an effective methodology but was less accurate than SVR. However, SVR was less accurate when the data became highly collinear [199].

### 7.2. Principal Component Analysis

PCA is used as predictive modelling tool in correlating large sets of data. PCA reduces the dimensionality of the data and minimizes data loss by the creation of uncorrelated variables the maximize variance [200]. The PCA transforms the current set of data to a new coordinate system. Then, a covariance matrix is developed, which, in turn, determines the eigenvectors and eigenvalues. These eigenvalues are then used to classify the data.

PCA is a common data analysis method in plant disease detection. The PCA method was used in the detection of diseases in the Catharanthus roseus leaves, which highlighted the benefits of PCA as an unsupervised clustering method that does not require a priori data set knowledge [52]. Research conducted on greenhouse pepper plant diseases using MSI found that PCA was not as effective in binary plant health detection and required implementation of a method using a priori knowledge [62]. This discrepancy in performance was blamed on the small colour difference between healthy and diseased leaves, since PCA is reliant on the variance of the data.

### 7.3. Self-Organizing Maps

A Self-Organizing Map (SOM) is an Artificial Neural Network (ANN) that takes high dimensional data and transforms it into a 2D map that places similar data points closer together [201]. As in most ANN, the SOM operates in a training mode dependent on training sample data sets and a mapping mode that converts new inputs into the visible map space. The quality of the training data set in similarity to the expected types of data determines the efficacy of SOM. The SOM is a rectangular grid which uses an iterative process to move the nodes of the grid toward the data where the grid moves toward the training data. This process is defined iteratively, where the Euclidean distance equation determines the similarity between points and weight vector where the best matching unit is found, and the weight vector is iteratively updated.

(24) $$W_{v}\left(s+1\right)=\;W_{v}\left(s\right)+\;\theta \left(u,v,s\right)\cdot \alpha \left(s\right)\cdot \left(D\left(t\right)-W_{v}\left(s\right)\right)$$

where *W_v_* is the weight vector, *s* is the iteration, *θ* is the neighbour function, *α* is a learning restraint and *D(t)* is input vector.

SOM has been used in data fusion applications to reduce the disease classification error of yellow rust in wheat using MSI and HSI sensors [86]. The analysis was also used to categorize grape leaf colours as a pre-processing technique to aid support vector machines (SVM) to then classify between three types of disease [202].

### 7.4. Artificial Neural Networks

ANN is a supervised learning technique that consists of a neuron net structure originally aimed at mimicking human brain problem-solving. Due to its structure, ANN is used to infer functions from the observed data, which learns a pathway to the output. In this example, the input can take multiple pathways to output with the hidden component, a weighted function (usually sigmoid) that uses a priori knowledge. The inputs, p, to the neuron are multiplied by a weight, w, the sum making the bias weight, then passed through a transfer function *σ*(*γ*) to obtain the output.

(25) $$\sigma \left(\gamma \right)=\frac{1}{\left(1+e^{-\gamma }\right)}$$

(26) $$\sigma \left(\gamma \right)\to \{\begin{array}{c} 1\;when\;\gamma \to \;+\infty \\ 0\;when\;\gamma \to \;-\infty \end{array}$$

ANN is used in the classification of fungal disease and other common anomalies in rice plants [68,203], the estimation of phosphorus content in cucumber leaves [55] and in the general image classification of the healthy and unhealthy leaves [204].

### 7.5. Support Vector Machines

**Figure 13:**
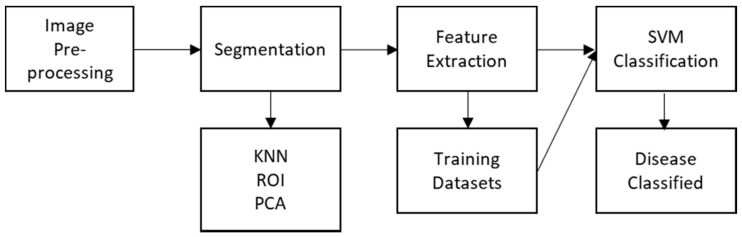
Support vector machines (SVM) in image-based data analysis adapted from [205].

Along with ANN, SVM is a typical algorithm used to classify plant diseases from the feature extraction process depicted in Figure 13.

SVM is a supervised technique that uses given sets data of at least two classes. With each new data set, the SVM measures the distance between the new points and the given data to classify it. A main variant of SVM is hard margin classifiers, which are especially useful in multi-dimensional data applications such as HSI and MSI. The SVM classification uses parallel hyperplanes based on the biggest margin between the hyperplanes.

(27) $$y_{i}\left(w,x_{i}-b\right)>1\;\mathrm{for}\;i=1\;to\;n\;\mathrm{and}\;\min\left|\left|w\right|\right|$$

where *b* is the bias, *i* indexes the *n* training data and *w* is normal to the hyperplane.

SVM has been widely adopted in the literature due to its proven performance in highly dimensional problems with small data sets [206]. The authors of [206] studied SVM in comparison to ANN classification, and found a significant improvement in plant disease classification for SVM over ANN [206]. SVM is used consistently in plant disease detection classification, with integration into genetic algorithms to improve the accuracy of the classification as suggested by the authors of [207].

### 7.6. K-Nearest Neighbours

K-Nearest Neighbours (KNN) is a widely used classification method with a learning algorithm based on the similarity of data points. Using a training dataset, once the algorithm makes the classification and groupings, the algorithm training is complete. This is a nonparametric algorithm, where the closeness to a number of known K points and voting process are used to classify the new data. Euclidean distance function is a common method to evaluate the closeness to the nearest points

(28) $$d=\;\sqrt{\overset{k}{\underset{i=1}{\sum }}{\left(x_{i}-y_{i}\right)}^{2}}$$

The KNN is simple and easy to implement. However, the method becomes inefficient when analysing large amounts of data.

### 7.7. Regions of Interest

Regions of interest (ROI) segmentation is used to define the borders or boundaries of an object and to describe an area in a compartmentalized way. ROI segmentation is essential for applying machine learning techniques for plant disease detection. In one study, researchers investigated the ROI applications based on colour variance between healthy and diseased parts of the plants [208]. The study found that the use of ROI techniques could achieve early disease detection at a rate of 91% and overcome the challenges of indistinct diseases boundaries. The use of ROI in the disease detection process, after image acquisition and some pre-processing, involves segmenting the images by means of ROI, which then leads to feature extraction and classification via means of SVM, as shown in Figure 5 [209]. This technique uses boundary and spot detection algorithms to locate an infected area, usually through K-Means clustering. From the clusters, the ROI are selected.

## 8. Conclusions

This review examined current and likely future electro-optical remote sensing applications for precision agriculture, with a focus on novel methods for early detection of plant diseases and the increasing adoption of spectral analysis in food quality assessment. The way in which plant diseases spread and cause subsequent deterioration of the plant determines the efficacy of each of the detection techniques discussed. For in situ applications, the sensitivity of thermography and fluorescence to ambient light reduces the accuracy and practicality of these techniques. Techniques reliant on spectral information must undergo a complex analysis to eliminate erroneous data due to the complex interactions with the environment. In consideration of the discussed techniques and their advantages and disadvantages, the evidence clearly points to a data fusion approach that combines multiple spectral techniques to improve the classification accuracy and to reduce the effects of external/environmental factors impacting the quality of data collected. Whereas the narrow band approach of HSI allows greater flexibility in distinguishing spectral areas of interest, the HSI sensor is particularly susceptible to erroneous data due to data overlap and is a costly/complex solution. The MSI system, in contrast, requires greater understanding of the spectral bands of interest before implementation. The added value of emerging LIDA-based systems is in their greater flexibility in capturing data as LIDAR systems are not as limited by atmospheric conditions, changes in light, viewing angle or canopy structure. The implementation of a low-cost, lightweight bistatic LIDAR system on a RS platform should be considered for future research, especially in the context of HSI-LIDAR data fusion. LIDAR is noted for its wide-ranging applications across all sensor platforms and, in combination with hyperspectral imaging, would likely be the most robust of the remote sensors. Driven by market demands, future work should be aimed toward food quality analysis using various combinations of remote sensors. The greatest hinderance for RS techniques is their indirect nature in correlating spectral or molecular data to a definitive diagnosis. The adoption of AI techniques for the spatially and temporally distributed big data acquired by RS is one of the biggest impacting factors on the accuracy of plant disease diagnosis. Comprehensive comparative studies of AI-based data fusion techniques in the precision agriculture context are needed to determine the best combination of sensors, platforms and crops, especially in the multi-sensor approach. Among the various sensor platforms, UAVs clearly fill a gap between the limitations of handheld and rover-carried sensors in large areas and of high-altitude and satellite sensor resolutions. The overall accuracy and resolution of UAV based sensors in precision agriculture should be further investigated, emphasising the disparities between leaf level and canopy level data acquisition. A multi-sensor approach, taking advantage of both traditional techniques and the emerging benefits of LIDAR-based in-field CO_2_ absorption measurements, is an area that requires further research to be able to improve early plant disease detection, soil analysis, phenotyping and fruit quality analysis. These multi-sensor systems will adopt AI-based data fusion algorithms to efficiently and accurately process the variety of spatially and temporally distributed data acquired in the field.

## Figures and Tables

**Table 1:** Typical abiotic and biotic stress factors.

Abiotic Factors	Biotic Factors
Nutrients	Fungi
Pesticides	Bacteria
Pollution	Nematodes
Temperature	Parasitic Plants
Light	Virus

**Table 2:** Common vegetation indices, adapted from [8].

Index	Equation	Plant Property
Normalized difference vegetation index (NDVI)	$\mathrm{NDVI}=\frac{R_{800}-R_{670}}{R_{800}+R_{670}}$	Biomass, leaf area
Simple ratio	$\mathrm{SR}=\frac{R_{800}}{R_{670}}$	Biomass, leaf area
Structure insensitive vegetation index	$\mathrm{SIPI}=\frac{R_{800}-R_{445}}{R_{800}+R_{680}}$	Ratio of carotenoids to chlorophyll
Pigments specific simple ratio	$\mathrm{PSSRa}=\frac{R_{800}}{R_{680}}$	Chlorophyll content
Anthocyanin reflectance index	$\mathrm{ARI}=\left(\frac{1}{R_{550}}\right)-\left(\frac{1}{R_{700}}\right)$	Anthocyanin
Red edge position	$\mathrm{REP}=700+\frac{40\left(R_{\mathrm{RE}}-R_{700}\right)}{\left(R_{740}-R_{700}\right)}$ $R_{\mathrm{RE}}=\frac{R_{670}+R_{780}}{2}$	Inflection point red edge

Reproduced with permission from Elsevier [8].

**Table 3:** Sample research on plant pathogen detection utilizing fluorescence spectroscopy.

Plant	Disease	Optimal Spectral Range	Data Analysis	Classification Accuracy ^1^	Reference
Orange trees	Citrus canker	442, 532 nm	Figure of merit		[50]
Greenhouse plants of Citrus limonia	Citrus canker	532 nm	Figure of merit		[51]
Catharanthus roseus LG Don	Infected by 10 types of phytoplasmas		PCA, Multivariate Data Analysis		[52]
Citrus leaves	Citrus canker	350–580 nm	Figure of merit	94–95%	[53]
Wheat	Yellow Rust	550–690 nm	Quadratic Discriminant Analysis (QDA)	99% (in conjunction with MSI)	[54]
Orange tree	Huanglongbing	Excitation: 405 nm Emission: 200–900 nm	PLSR	>90%	[45]
Cucumber Leaves	N/A (Phosphorus)	325–1075 nm	PLSR, ANN, SVM	75%	[55]
Ziziphus	Quercetin	350–800 nm	PLSR		[56]
Soybeans	N/A (phenotyping)	Excitation: 405 nm Emission: 194–894 nm	Regression and PLSR	85–96%	[24]

^1^ Classification accuracy is dependent on the scope of the research and time of testing.

**Table 4:** Summary of parameters from sensors [59]. (CC BY 4.0).

Sensor	Index	Equation	Indicator
Thermography	Maximum temperature difference	MTD = max − min temperature	Biotic stresses in early stage
	Average temperature difference	ΔT = average air temperature − average measured temperature	Biotic stresses in early and late stages
Chlorophyll fluorescence imaging	Maximal fluorescence yields	Fm	Fast chlorophyll fluorescence kinetics
	Maximal PSII quantum yields (Fv/Fm)	Fv/Fm = (Fm − F_0_)/Fm	Maximal photochemical efficacy of photosynthesis II
	Effective PSII quantum yield (Y[II])	Y[II] = (Fm’ − F)/Fm’	Photochemical quantum yields at steady state
Hyperspectral Imaging	Normalized differences vegetation index (NDVI)	NDVI = (R_800_ − R_670_)/(R_800_ + R_670_)	Biomass, leaf area
	Photochemical reflection index (PRI)	PRI = (R_531_ − R_570_)/(R_531_ + R_570_)	Pigments, photosynthetic efficiency
	Pigment-specific simple ration (PSSR)	PSSRa = R_800_/R_680_	Chlorophyll a
		PSSRb = R_800_/R_635_	Chlorophyll b
		PSSRc = R_800_/R_470_	Carotenoid
	Water Index (WI)	WI = R_900_/R_970_	Water content

**Table 5:** Sample research on plant pathogen detection utilizing multispectral imaging.

Plant	Disease	Optimal Spectral Range	Data Analysis	Classification Accuracy ^1^	Reference
Avocado	Laurel wilt (LW)	10–580, 10–650, 10–740, 10–750, 10–760 and 40–850 nm	Multilayer perceptron (MLP) and Radial basis function (RBF)		[61]
Bell pepper	Powdery mildew (PM) and Tomato spotted wilt virus (TSWV)	520–920 nm	Principal component analysis (PCA), Linear discriminant analysis (LDA) and Quadratic discriminant analysis (QDA)		[62]
Cassava (Manihot esculenta Crantz)	Cassava Mosaic virus Disease (CMD)	531 and 570 nm	Regions of interest (ROI), Receiver operating characteristic (ROC)		[63]
Cassava (Manihot esculenta Crantz)	Cassava Mosaic virus Disease (CMD)	684, 687, 757.5, 759.5 nm	Fraunhofer line discrimination (FLD), Pseudo-colour mapped (PCM) and Regions of interest (ROI)		[64]
Grapevine	Powdery mildew (PM)	540, 660, 800 nm	Regions of interest (ROI)		[65]
Creeping bentgrass (turfgrass)	Rhizoctonia solani	760–810 nm	Linear Regression Analysis	<50%	[66]
Wheat	Yellow Rust	861, 543 nm	Self-Organising Maps (SOM), MANOVA	95%	[67]
Rice plants	Snails	N/A	ANN	91%	[68]
Grapevines	Flavescence dorée	455–495, 540–580, 658–678, 707–727, 800–880 nm	Pix4D software, univariate and multivariate classification approach, RMSE	80–90%	[69]

^1^ Classification accuracy is dependent on the scope of the research.

**Table 6:** Comparison of remote sensing imaging techniques adapted [76]. (CC BY 4.0).

Features	Molecular Methods	Fluorescence Spectroscopy	Multispectral Imaging	Hyperspectral Imaging	LIDAR
Spatial information			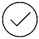	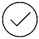	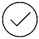
Spectral information		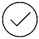	Limited	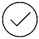	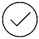
Sensitive to minor components	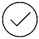	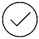	Limited	Limited	Limited
Building chemical images	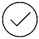	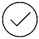	Limited	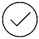	Limited
Flexibility of spectral information extraction		Limited	Limited	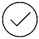	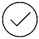

**Table 7:** Sample research of plant pathogen detection utilizing hyperspectral imaging.

Plant	Disease	Optimal Spectral Range	Data Analysis	Reference	Classification Accuracy ^1^
Cotton	Herbicide drift	325–1075 nm	Partial least squares regression (PLSR)	[89]	
Cotton	Verticillium wilt	620–700, 1001–1110, 1205–1320 nm	Severity Level (SL)	[90]	
Strawberry	Anthracnose crown rot (ACR)	350–2500 nm	Fisher discriminant analysis (FDA), Stepwise discriminate analysis (SDA) and k-nearest neighbour (kNN)	[91]	
Tulips	Tulip breaking virus (TBV)	430–900 nm	Fisher’s linear discriminant analysis (LDA)	[92]	
Oil palms	Ganoderma	460–959 nm	One-way ANOVA	[93]	
Oil Palms	Ganoderma	430–900 nm	Lagrangian interpolation, MNF	[94]	73–84%
Soybean		800 nm	Image intensity data (1-y)	[9]	
Grapefruit	Citrus canker	450–930 nm	SDK, SID	[95]	96%
Apple trees	Venturia inaequalis (apple scab)	1350–1750, 2200–2500, 650–700 nm	Logistic regression, PLSR, logistic discriminant analysis, and tree-based modelling	[96]	
Rice plants	Brown planthopper and leaf folder infestations	445, 757 nm	Linear correlation	[97]	R = 0.92
Wheat	Yellow Rust	550–690 nm	Kohonen maps, Self-Organizing maps, QDA	[86]	99%
Barely	N/A (Nitrogen and phosphorus content)	450–700 nm	PLSR	[98]	75%
Apples	Apple bruises/fungal	430–930 nm		[99]	

^1^ Classification accuracy is dependent on the scope of the research.

**Table 8:** Fluorescence- and carbon dioxide-based models of plant health.

Parameter	Fluorescence Model	CO_2_ Model
Irradiance	Solar	Laser Emitter
Radiance	Canopy	Noise
Spectrum Window Interval	Oxygen absorption 680–698 nm 750–780 nm	CO_2_ absorption cross-section 1568–1675 nm
Function-based	Gaussian	Beer Lambert
Measured object	Small amount of fluorescence signal from background reflectance	Molecular absorption within transmitted beam

**Table 9:** Comparison of remote sensing platforms [183]. (CC BY 4.0).

		UAV	Aerial	Satellite
Mission	Range	Poor	Good	Optimal
	Flexibility	Optimal	Good	
	Endurance	Poor	Optimal	Optimal
	Cloud cover dependency	Optimal	Good	Poor
	Reliability	Average	Good	Optimal
Processing	Payload	Average	Good	Optimal
	Resolution	Optimal	Good	Average
	Precision	Optimal	Good	Average
	Processing time	Average	Good	Good

**Table 10:** Comparison of remote sensing platforms using multispectral imaging for detection of grape disease [183]. (CC BY 4.0).

Platform	Spectral Wavelength	Altitude	Resolution (Pixels)
UAV	520–600, 630–690, 760–900 nm	150 m	2048 × 1536
Aerial	415–425, 526–536, 545–555, 565–575, 695–705, 710–720, 745–755, 490–510, 670–690 770–790, 790–810, and 890–910 nm	2300 m	2048 × 2048
Satellite	440–510, 520–590, 630–680, 690–730, and 760–850 nm	630 km	12,000 (pixel linear CCD per band)
